# Long-term outcomes of severe rheumatic mitral stenosis after undergoing percutaneous mitral commissurotomy and mitral valve replacement: A 10-year experience

**DOI:** 10.34172/jcvtr.2022.16

**Published:** 2022-06-12

**Authors:** Wasinee Promratpan, Nonthikorn Theerasuwipakorn, Vorarit Lertsuwunseri, Suphot Srimahachota

**Affiliations:** Division of Cardiovascular Medicine, Department of Medicine, Faculty of Medicine, Chulalongkorn University, Cardiac Center, King Chulalongkorn Memorial Hospital, 10330, Bangkok, Thailand

**Keywords:** Long-Term Outcome, Mitral Valve Replacement, Percutaneous Mitral Commissurotomy, Rheumatic Mitral Stenosis

## Abstract

***Introduction:*** Percutaneous mitral commissurotomy (PTMC) and mitral valve replacement (MVR) are treatments of choice for severe rheumatic mitral stenosis (MS). Data regarding the long-term outcomes of patients who underwent PTMC and MVR are limited.

***Methods:*** A retrospective cohort study was conducted to evaluate the long-term outcomes of patients with severe rheumatic MS who underwent PTMC or MVR between 2010 to 2020. The primary outcome comprised of all-cause death, stroke or systemic embolism, heart failure hospitalization and re-intervention. Cox regression was used to investigate predictors of the primary outcome.

***Results:*** 264 patients were included in analysis, 164 patients (62.1%) in PTMC group and 100 patients in MVR group (37.9%). The majority were females (80.7%) and had atrial fibrillation (68.6%). The mean age was 49.52 (SD: 13.03) years old. MVR group had more age and AF, higher Wilkins’ score with smaller MVA. Primary outcome occurred significantly higher in PTMC group (37.2% vs 22%, *P*=0.002), as well as, re-intervention (18.3% vs 0%, *P*<0.001). However, all-cause mortality, stroke or systemic embolism and heart failure hospitalization were not significantly different. In multivariate Cox regression analysis, PTMC (HR 1.94; 95%CI 1.14, 3.32; *P*=0.015), older age (HR 1.03; 95%CI 1.01, 1.06; *P*=0.009) and SPAP > 50 mmHg (HR 2.99; 95%CI 1.01, 8.84; *P*=0.047) were the only predictors of primary outcome.

***Conclusion:*** Primary outcome occurred in PTMC group more than MVR group which was driven by re-intervention. However, all-cause mortality, stroke or systemic embolism and heart failure hospitalization were not significantly different.

## Introduction

 Rheumatic heart disease (RHD) is one of the most common acquired valvular heart diseases. ^1^ RHD has declined dramatically worldwide, though in low- to middle-income countries, RHD is an important cause of death and disability. The prevalence of RHD ranged from 3 to > 1,000 cases per 100,000 depending on regional endemic. ^2,3^ Mitral stenosis (MS), the most common manifestation of RHD, can cause atrial fibrillation (AF), ischemic stroke, pulmonary hypertension, and heart failure. The treatment strategies for clinically significant rheumatic MS are percutaneous mitral commissurotomy (PTMC) and mitral valve replacement (MVR). PTMC is the treatment of choice in patients with favorable clinical and valvular anatomical characteristics while some patients with contraindication to PTMC should undergo MVR. ^4,5^

 Treatment results are variable depending on many factors including patient and mitral valve (MV) characteristics, as well as, the local expertise of interventionists and surgeons.^6-10^Moreover, long-term outcomes of patients with severe rheumatic MS who underwent PTMC or MVR are limited. The aims of this study are to evaluate long-term outcomes, procedural success rate and complications of these patients.

## Materials and Methods

###  Study design

 This is a single-center retrospective cohort study conducted in patients with age ≥ 18 years old and diagnosed of a clinically significant severe rheumatic MS, mitral valve area (MVA) < 1.5 cm^2^, who underwent either PTMC or MVR including MVR with tricuspid valve repair (TVR) during 2010 to 2020. Patients who had inadequate follow-up time (< 6 months), indication for other cardiac surgery or previously underwent mitral valve intervention were excluded. The patient’s information was reviewed from OPD records, IPD records and civil registration. This study was approved by the Institutional Review Board (IRB no.672/63).

###  Procedures

 The treatment strategy, including the prosthetic valve types (bioprosthesis or mechanical valve) and the need for concomitant tricuspid valve annuloplasty (TVA) in case of MVR, was decided by the heart team which consisted of cardiothoracic surgeons, cardiologists, echocardiographic specialists and anesthesiologists.

 PTMC was performed with the Inoue commissurotomy technique using Inoue single balloon (Toray Industries, Inc., NY, United State) and transesophageal guided atrial septostomy and commissurotomy. ^11^ A balloon diameter and catheter size were chosen according to the patient height. Echocardiography, as well as left and right cardiac catheterization, were performed at baseline and after PTMC. Important parameters namely MVA, mean pressure gradient (PG) across MV, Wilkins’ score, mitral regurgitation (MR) grading and pulmonary artery pressure were recorded.

###  Outcomes 

 The primary outcome was composite of all-cause death, stroke or systemic embolism, heart failure hospitalization and re-intervention rate. Secondary outcomes were all-cause death, stroke or systemic embolism, heart failure hospitalization, re-intervention rate, PTMC success rate, periprocedural complications, valvular infection and serious bleeding (The Bleeding Academic Research Consortium (BARC) definition type 3 or more). ^12^ PTMC success rate was defined as MVA after procedure > 1.5 cm^2^ or more than twice of the preprocedural value and no worsening of MR more than grade 2+). ^13^

###  Statistical analysis 

 Categorical variables were presented as frequency and percentage and analyzed using a Chi-square test or Fisher’s exact test as appropriate. Continuous variables are presented as the mean with standard deviation (SD) or median with interquartile range (IQR) and analyzed using a t-test or Mann-Whitney test as appropriate. The periprocedural complications were not analyzed due to the different complications found between both groups. Univariate and multivariate Cox regression, adjusted for covariates with a p-value from the univariable model was less than 0.15, were performed to find the hazard ratio (HR). The Kaplan-Meier curve with log-rank tests was used for survival analysis. All analyses required a value of p < 0.05 for statistical significance. All statistical analyses were performed using SPSS Statistics version 22.0 (IBM Corp., Armonk, NY, USA) and STATA/SE version 14.1 (StataCorp., Texas, USA).

## Results

###  Baseline characteristics 

**Figure 1:**
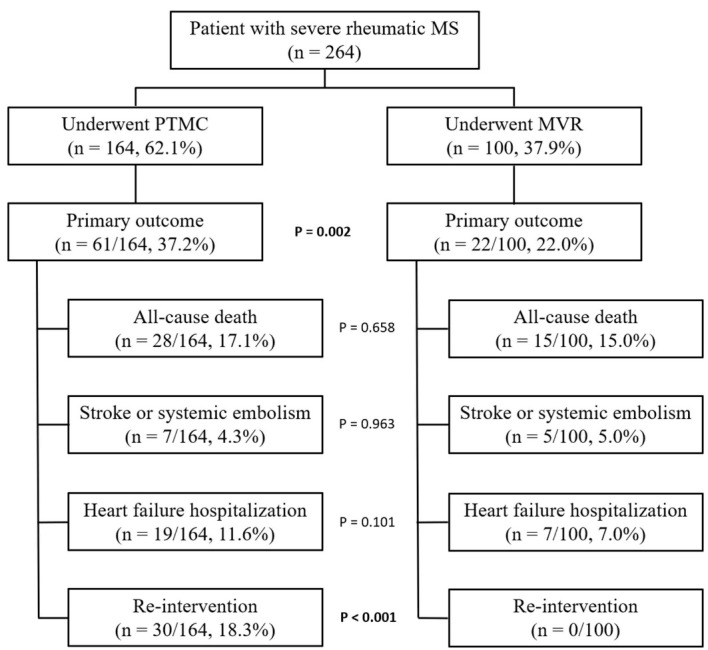
Outcomes of patients with severe rheumatic MS who underwent PTMC and MVR Bold value denotes statistical significance (P < 0.05). MS = Mitral stenosis, MVR = Mitral valve replacement, PTMC = Percutaneous mitral commissurotomy.

 Two hundred and sixty-four patients were included in the analysis, 164 patients (62.1%) in the PTMC group and 100 patients in the MVR group (37.9%) (Figure 1). The majority were females (80.7%) and had AF (68.6%). The mean age was 49.52 (SD: 13.03) years old. Hypertension (HT), type 2 diabetes mellitus (DM), dyslipidemia and chronic kidney disease (CKD) were found 14.4%, 10.2%, 6.8%% and 1.9%, respectively. The most common indications for MV intervention were dyspnea (50.8%), heart failure (37.1%) and new-onset AF (14.4%) (Table 1).

**Table 1:** Baseline characteristics

	**All patients (N=264)**	**PTMC group (N=164)**	**MVR group (N=100)**	***P*** ** value**
**Mean age**, years	49.52 ± 13.03	47.38 ± 13.38	53.04 ± 11.69	0.001
**Female,** (%)	213 (80.7%)	131 (79.9%)	82 (82%)	0.672
**Atrial fibrillation,** (%)	181 (68.6%)	87 (53%)	94 (94%)	< 0.001
**Comorbidity, **(%)				
Type 2 diabetes mellitus	27 (10.2%)	18 (11%)	9 (9%)	0.626
Hypertension	38 (14.4%)	24 (14.6%)	14 (14%)	0.912
Dyslipidemia	18 (6.8%)	9 (5.5%)	9 (9%)	0.262
Chronic kidney disease, (GFR < 60 ml/min/1.73 m^2^)	5 (1.9%)	4 (2.4%)	1 (1%)	0.653
**Indication**, (%)				
Dyspnea	134 (50.8%)	84 (51.2%)	50 (50%)	0.897
Heart failure	98 (37.1%)	56 (34.1%)	42 (42%)	0.181
Stroke and systemic embolism	30 (11.4%)	18 (11%)	12 (12%)	0.782
New onset atrial fibrillation	38 (14.4%)	29 (17.7%)	9 (9%)	0.054
SPAP > 50 mmHg	8 (3%)	6 (3.7%)	2 (2%)	0.714
Planned pregnancy or major surgery	9 (3.4%)	9 (5.5%)	0 (0%)	0.018
**Preprocedural Echocardiographic data**				
Wilkins’ score	8.59 ± 1.85	8.10 ± 1.56	9.47 ± 2.01	< 0.001
Wilkins’ score ≥ 8, (%)	192 (72.7%)	112 (68.3%)	80 (80%)	< 0.001
MVA by planimetry, cm^2^	0.91 ± 0.30	0.94 ± 0.27	0.85 ± 0.32	0.016
MVA by PHT, cm^2^	0.96 ± 0.28	0.98 ± 0.26	0.89 ± 0.27	0.005
Mean PG, mmHg	12.67 ± 5.81	12.74 ± 6.11	12.56 ± 5.5	0.823
RVSP, mmHg	54.77 ± 22.77	54.19 ± 23.10	55.70 ± 21.23	0.631
Mean PAP, mmHg	32.83 ± 12.00	34.48 ± 13.50	31.58 ± 11.27	0.269
Follow up time*,month	62.0 (28.0, 102.0)	62.5 (27.25, 101.0)	57.0 (28.25, 102.75)	0.966

* Median with interquartile range GFR = Glomerular filtration rate, MVA = Mitral valve area, MVR = Mitral valve replacement, PAP = Pulmonary arterial pressure, PHT = Pressure-half time, PG = pressure gradient, PTMC = Percutaneous mitral commissurotomy, RVSP = Right ventricular systolic pressure, SPAP = Systolic pulmonary arterial pressure.

 In the PTMC group, the mean age was 47.38 (SD: 13.38) years old and 53% had AF, significant lower when compared to the MVR group whose mean age was 53.04 (SD: 11.69) years old (*P* = 0.001) and 94% had AF (*P* < 0.001). The comorbidities and indications for MV intervention in both groups were comparable. The only different indication was intervention before pregnancy or undergoing major surgery which only led to PTMC (5.5%) but not surgery (*P* = 0.018). In term of echocardiographic parameters, MVR group had more severe MV morphology: mean Wilkins’ score was 9.47 (SD: 2.01) vs 8.1 (SD: 1.56), *P* < 0.001; mean MVA using planimetry was 0.85 (SD: 0.32) vs 0.94 (SD: 0.27), *P* = 0.016; mean MVA using pressure half time (PHT) was 0.89 (SD: 0.27) vs 0.98 (SD: 0.26), *P* = 0.005. However, mean PG across MV and estimated right ventricular systolic pressure (RVSP) was not significantly different. The median follows up time was 62.5 (IQR: 27.25, 101.0) months in the PTMC group and 57.0 (IQR: 28.25, 102.75) months in the MVR group which were comparable in both groups (Table 1).

###  Treatment outcomes

####  PTMC group

 The primary outcome occurred in 61 patients (37.2%), consisted of all-cause mortality 17.1%, stroke or systolic embolism 4.3%, heart failure hospitalization 11.6% and re-intervention 18.3% (Figure 1). The success rate of PTMC was 67.1%, however, periprocedural complications occurred in 14 patients (8.5%) including cardiac tamponade in 5 patients (3%), severe MR in 8 patients (4.9%) and 1 death (0.6%). The valvular infection and serious bleeding (BARC ≥ 3) were 1.2% and 8.5%, respectively. The median length of hospital stays was 1 (IQR: 1, 2) days (Table 2). During the follow-up, 30 patients (18.3%) were undergoing re-intervention with a median intervention-free period of 40.0 (IQR: 10.0, 77.5) months. The indications for re-intervention were severe MS 70% and severe MR 30%. Re-intervention was done with PTMC in 7 patients (23.3%) and MVR in 23 patients (76.7%) as shown in the Supplementary Table (Online resource 1).

**Table 2:** Outcomes of PTMC and MVR groups

	**PTMC group** **(N,164)**	**MVR group** **(N,100)**	***P*** ** value**
**Primary outcome: **all-causedeath; stroke or systemic embolism; heart failure hospitalization and re-intervention; (%)	61 (37.2%)	22 (22%)	0.002
**All-causes mortality**;(%)	28 (17.1%)	15 (15%)	0.658
**Stroke or systemic embolism**; (%)	7 (4.3%)	5 (5%)	0.963
**Heart failure hospitalization**; (%)	19 (11.6%)	7 (7%)	0.101
**Re-intervention**; (%)	30 (18.3%)	0 (0%)	< 0.001
**Periprocedural complication**; (%)	14 (8.5%)	16 (16%)	N/A
Cardiac tamponade	5 (3%)	0	
Severe MR	8 (4.9%)	0	
Re-sternotomy	N/A	4 (4%)	
AKI required dialysis	0	4 (4%)	
Complete heart block			
- Temporary pacemaker	0	3 (3%)	
- Permanent pacemaker	0	1 (1%)	
Death	1 (0.6%)	4 (4%)	
**Valvular infection**; (%)	2 (1.2%)	5 (5%)	0.911
**Bleeding**; (%)	14 (8.5%)	12 (12%)	
**Severity **(according to BARC definition)			
- Non serious bleeding (BARC < 3)	11 (6.7%)	5 (5%)	Reference
- Serious bleeding (BARC ≥ 3)	3 (1.8%)	7 (7%)	0.062
**Site of serious bleeding**			
- Intracranial hemorrhage	1 (0.6%)	4 (4%)	
- GI tract bleeding	2 (1.2%)	0	
- Joint and muscle bleeding	0	2 (2%)	
- Others	0	1 (1%)	
Length of hospital stays*; days	1 (1; 2)	9 (9; 16)	< 0.001

Abbreviations: AKI, Acute kidney injury; BARC , Bleeding Academic Research Consortium; MR , Mitral regurgitation; MVR , Mitral valve replacement; PTMC , Percutaneous mitral commissurotomy. *Median with interquartile range

 After PTMC, MVA by planimetry (0.94, SD: 0.27 vs 1.49 SD: 0.39, *P* < 0.001) and MVA by PHT (0.98, SD: 0.26 vs 1.56 SD: 0.38, *P* < 0.001) were significantly improved. Mean PG across MV measured with echocardiography (12.74 SD: 6.11 vs 6.12 SD: 2.9, *P* < 0.001) and cardiac catheterization (12.63 SD: 6.71 vs 5.87 SD: 3.79, *P* < 0.001) was decrease by a half. RVSP, systolic pulmonary artery pressure (PAP) and mean PAP were also decrease significantly (*P* < 0.001 for all parameters) (Table 3).

**Table 3:** Echocardiographic and cardiac catheterization parameters at baseline and after PTMC

	**Preprocedural**	**Post procedural**	***P*** ** Value**
**Echocardiographic data**			
MVA by planimetry, cm^2^	0.94 ± 0.27	1.49 ± 0.39	< 0.001
MVA by PHT, cm^2^	0.98 ± 0.26	1.56 ± 0.38	< 0.001
Mean PG, mmHg	12.74 ± 6.11	6.12 ± 2.9	< 0.001
RVSP, mmHg	54.78 ± 23.10	44.42 ± 18.50	< 0.001
**Cardiac catheterization**			
Mean PG, mmHg	12.63 ± 6.71	5.87 ± 3.79	< 0.001
SPAP, mmHg	61.74 ± 21.43	49.53 ± 17.19	< 0.001
Mean PAP, mmHg	40.43 ± 12.94	31.66 ± 11.32	< 0.001

Abbreviations: MVA , Mitral valve area; PAP , Pulmonary arterial pressure; PHT , Pressure-half time; PG , pressure gradient; PTMC , Percutaneous mitral commissurotomy; RVSP , Right ventricular systolic pressure; SPAP , Systolic pulmonary arterial pressure.

####  MVR group

 Patients with contraindication to PTMC were undergoing MVR. Contraindications were the presence of left atrial thrombus (22%), at least moderate MR (43%), severe tricuspid regurgitation requiring surgery (26%), unfavorable MV characteristics (13%), severe bi-commissural fusion (1%), the absence of commissural fusion (1%).

 Of all patients in the MVR group, MVR alone was done in 72% while 28% underwent MVR and concomitant TVA. Seventy-four patients (74%) were implanted with a mechanical valve and 26 patients were implanted with a bioprosthetic valve.

 The primary outcome occurred in 22 patients (22%), consisting of all-cause mortality 15%, stroke or systolic embolism 5% and heart failure hospitalization 7%. There was no re-intervention in this group (Figure 1). The periprocedural complications occurred in 16 patients (16%) including re-sternotomy (4%), acute kidney injury required hemodialysis (4%), complete heart block (4%) and death (4%). The valvular infection and serious bleeding (BARC ≥ 3) were 5% and 12%, respectively. The median length of hospital stays was 9 (IQR: 9, 16) days (Table 2).

###  Primary and secondary outcomes

 Primary outcome occurred significantly higher in PTMC group (37.2% vs 22%, p = 0.002), as well as, re-intervention (18.3% vs 0%, p < 0.001). However, all-cause mortality (17.1% vs 15%, p = 0.658), stroke or systemic embolism (4.3% vs 5%, p = 0.963), heart failure hospitalization (11.6% vs 7%, p = 0.101), valvular infection (1.2% vs 5%, p = 0.911) and serious bleeding (1.8% vs 7%, p 0.062) were not significantly different.

###  Cox regression and Kaplan-Meier survival analyses

 Potential predictors from univariable analysis were MV intervention with PTMC, older age, DM, HT and SPAP > 50 mmHg as an indication for MV intervention. After adjusted with potential confounding covariates in multivariable analysis; however, PTMC (HR 1.94; 95% confident interval (CI) 1.14, 3.32; p = 0.015), older age (HR 1.03; 95% CI 1.01, 1.06; p = 0.009) and SPAP > 50 mmHg (HR 2.99; 95% CI 1.01, 8.84; p = 0.047) were only predictors of primary outcome (Table 4).

**Table 4:** Univariate and multivariate cox regression analysis for primary outcome

	**Univariate analysis**			**Multivariate analysis**		
	**HR**	**95% CI**	**p-value**	HR*	**95% CI**	***p*** **-value**
**PTMC**	1.71	1.04, 2.79	0.034	1.94	1.14, 3.32	0.015
**Age**	1.04	1.02, 1.06	<0.001	1.03	1.01, 1.06	0.009
**Female**	0.94	0.55, 1.60	0.815			
**Atrial fibrillation**	1.42	0.86, 2.32	0.168			
**Comorbidity**						
Type 2 diabetes mellitus	3.64	2.18, 6.09	<0.001	2.29	1.00, 5.25	0.050
Hypertension	2.19	1.32, 3.65	0.003	0.98	0.45, 2.16	0.960
Dyslipidemia	1.51	0.76, 3.03	0.244			
Chronic kidney disease, (GFR < 60 ml/min/1.73 m^2^)	2.72	0.85, 8.67	0.091	1.80	0.54, 6.03	0.343
**Indication**						
Dyspnea	0.70	0.45, 1.09	0.113	0.76	0.46, 1.26	0.287
Heart failure	1.37	0.88, 2.13	0.170			
Stroke and systemic embolism	0.98	0.50, 1.90	0.949			
New-onset atrial fibrillation	1.04	0.58, 1.85	0.905			
SPAP > 50 mmHg	3.15	1.13, 8.76	0.028	2.99	1.01, 8.84	0.047
Planned pregnancy or major surgery	0.75	0.10, 5.44	0.778			
**Preprocedural Echocardiographic data**						
Wilkins’ score	1.07	0.95, 1.21	0.270			
Wilkins’ score ≥ 8, (%)	1.16	0.66, 2.05	0.599			
MVA by planimetry, cm^2^	0.57	0.24, 1.35	0.199			
Mean PG, mmHg	0.96	0.92, 1.01	0.107	1.00	0.95, 1.05	0.938
RVSP, mmHg	1.01	0.99, 1.02	0.249			
Mean PAP, mmHg	1.01	0.98, 1.03	0.559			

Abbreviations: GFR , Glomerular filtration rate; MVA , Mitral valve area; PAP , Pulmonary arterial pressure; PG , pressure gradient; PTMC , Percutaneous mitral commissurotomy; RVSP , Right ventricular systolic pressure; SPAP , Systolic pulmonary arterial pressure. * Adjusted with factors which p-value in univariate analysis < 0.15

**Figure 2:**
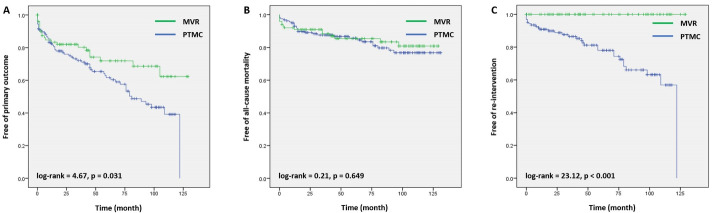
Kaplan-Meier analysis curves for **(A)** primary composite outcome, **(B)** all-cause mortality and **(C)** re-intervention according to strategy of mitral valve intervention (PTMC vs MVR). MVR = Mitral valve replacement, PTMC = Percutaneous mitral commissurotomy.

 From the Kaplan-Meier curve, MV intervention with PTMC had a significant higher rate of primary outcome (log-rank 4.67; p = 0.031) and re-intervention rate (log-rank 23.12; p < 0.001) than MVR but not for the all-cause mortality (log-rank 0.21; p = 0.649) (Figure 2A-C).

## Discussion

 Unlike most of the previous studies which studied on PTMC or MVR alone, this study evaluated long-term outcomes of patients with clinically significant severe rheumatic MS who underwent MV intervention either PTMC or MVR within 10 years period. We found that the primary composite outcome comprised of all-cause death, stroke or systemic embolism, heart failure hospitalization and re-intervention was significantly higher in the PTMC group (37.2% vs 22%, p = 0.002). The higher primary outcome in the PTMC group was driven by the incidence of re-intervention (18.3% vs 0%, p < 0.001). However, all-cause death, stroke or systemic embolism and heart failure hospitalization were not significantly different between the two groups. Previous studies reported a wide range of long-term outcomes depending on patient characteristics in each study. All-cause mortality was reported ranging from 0.6 – 14% after PTMC and 6 - 25% after MVR. ^6,7,9,14-16^ In this study, all-cause mortality was 17.1% after PTMC and 15% after MVR, supporting the results of the previous studies. In addition, stroke and systemic embolism rate (4 - 5%) was similar to the previous reports (2 – 4%). ^14,16^

 Regarding survival analyses, we found that MV intervention with PTMC (HR 1.94; 95% CI 1.14, 3.32; p = 0.015), older age (HR 1.03; 95% CI 1.01, 1.06; p = 0.009) and SPAP > 50 mmHg as an indication for MV intervention (HR 2.99; 95% CI 1.01, 8.84; p = 0.047) increased risk of primary outcome.

 After PTMC, the mean MVA was increased by 0.58 cm^2^ which was less than the previous report (0.84 cm^2^) and the success rate was lower (67.1% vs 80-95%). ^17,18^ However, preprocedural MVA in current study was smaller than the previous report by 0.15 cm^2^. Besides, there was a significant proportion (68.3%) of patients with Wilkins’ score ≥ 8 in this study, while excluded by previous studies. To our knowledge, MVA before intervention and Wilkins’ score were important predictors of PTMC results. ^19^ Defined by postprocedural MVA > 1.5 cm^2^, many patients were classified as unsuccessful PTMC because MVA was not exceeding 1.5 cm^2^, although, their symptoms and MVA improved. Supported by the re-intervention rate in the current study was similar to other reports (18.3% vs 12 – 40%) and PTMC could delay further intervention by a median of 40.0 (IQR: 10.0, 77.5) months even in patients with Wilkins’ score ≥ 8, hence, unsuccessful PTMC by echocardiographic criteria might not be a good representative of clinical outcomes. ^14,15,20^

 When compared to PTMC, the MVR group had more age and AF, higher Wilkins’ score with smaller MVA indicated more disease severity and chronicity. The median length of hospital stay in the MVR group was 8-day longer than the PTMC group supported the result of the previous study. ^20^ Periprocedural complications including death were higher in the MVR group, however, long-term outcomes were not different.

 Due to a high proportion of patients with Wilkins’ score ≥ 8, this study showed evidence that PTMC could be considered and performed successfully in this patient group, especially when MVR was inappropriate or not preferred. Nevertheless, a prospective study should be further investigated to confirm the result.

 This study had several limitations. First, this was a retrospective study, therefore outcomes were prone to review bias and subject to confounding from other factors. Second, there was no cardiac catheterization data in the MVR group, hence we could not compare PAP after intervention between groups. Third, this study was conducted in a tertiary referral center where interventionists and surgeons were experienced, thus limiting its generalizability especially in patients with Wilkins’ score ≥ 8 and very small MVA < 1.0 cm^2^.

## Conclusion

 Primary composite outcome occurred in PTMC group more than MVR group which was driven by re-intervention. PTMC group had a higher re-intervention rate, though, it could postpone further invasive procedure by 40 months. Moreover, PTMC could be performed successfully in patients with Wilkins’ score ≥ 8 and might be considered particularly when a patient was not suitable for MVR. All-cause mortality, stroke or systemic embolism, heart failure hospitalization, valvular infection and serious bleeding were not significantly different between two groups.

## Acknowledgements

 We would like to thank all King Chulalongkorn memorial hospital catheterization and noninvasive laboratory staffs involved in this study.

## Funding

 None.

## Ethical approval

 This retrospective chart review study involving human participants was in accordance with the ethical standards of the institutional and national research committee and with the 1964 Helsinki Declaration and its later amendments or comparable ethical standards. The Human Investigation Committee (IRB) of Chulalongkorn University approved this study (IRB no.672/63).

## Competing interest

 All authors declare that they do not have any conflict of interest.

## Supplementary files

Supplementary file contains Table S1.
