# Supplementary material for: Long-term outcomes of severe rheumatic mitral stenosis after undergoing percutaneous mitral commissurotomy and mitral valve replacement: A 10-year experience
Source: J Cardiovasc Thorac Res. 2022 Jun 12;14(2):101–7. doi: 10.34172/jcvtr.2022.16 (PMC9339733; doi:10.34172/jcvtr.2022.16)
Supplement: Supplementary file 1 — Supplementary file contains Table S1. [file jcvtr-14-101-s001.pdf]

Table S1. Re-intervention data

**Re-intervention,** (%)	30 (18.3%)
**Intervention-free survival time*,** months	40.0 (10.0, 77.5)
**Timing,** (%)	
< 1 month	6 (20%)
1month – 1 year	5 (16.7%)
1-5years	10 (33.3%)
> 5 years	9 (30%)
**Indication,** (%)	
Mitral stenosis	21 (70%)
Mitral regurgitation	9 (30%)
**Procedure,** (%)	
PTMC	7 (23.3%)
MVR	23 (76.7%)

* Median with interquartile range

MVR = Mitral valve replacement, PTMC = Percutaneous mitral commissurotomy
